# VCAM-1 expression is upregulated by CD34^+^/CD133^+^-stem cells derived from septic patients

**DOI:** 10.1371/journal.pone.0195064

**Published:** 2018-03-30

**Authors:** Christian Patry, Christoph Remmé, Christian Betzen, Burkhard Tönshoff, Benito A. Yard, Grietje Beck, Neysan Rafat

**Affiliations:** 1 Department of Pediatrics I, University Children’s Hospital Heidelberg, Heidelberg, Germany; 2 Institute of Physiology and Pathophysiology, Division of Cardiovascular Physiology, University of Heidelberg, Heidelberg, Germany; 3 Department of Anaesthesiology and Critical Care Medicine, University Medical Center Mannheim, Mannheim, Germany; 4 Division of Functional Genome Analysis, German Cancer Research Center (DKFZ), Heidelberg, Germany; 5 Department of Medicine V, University Medical Centre Mannheim, Mannheim, Germany; 6 Department of Anaesthesiology and Critical Care Medicine, HELIOS Dr. Horst Schmidt Kliniken, Wiesbaden, Germany; 7 Department of Pharmaceutical Sciences, Bahá’í Institute of Higher Education (BIHE), Teheran, Iran; Centro Cardiologico Monzino, ITALY

## Abstract

CD34^+^/CD133^+^- cells are a bone marrow derived stem cell population, which presumably contain vascular progenitor cells and are associated with improved vascular repair. In this study, we investigated whether the adhesion molecules ICAM-1 (intercellular adhesion molecule-1), VCAM-1 (vascular adhesion molecule-1), E-selectin und L-selectin, which are involved in homing of vascular stem cells, are upregulated by CD34^+^/CD133^+^-stem cells from septic patients and would be associated with improved clinical outcome. Peripheral blood mononuclear cells from intensive care unit (ICU) patients with (n = 30) and without sepsis (n = 10), and healthy volunteers (n = 15) were isolated using Ficoll density gradient centrifugation. The expression of VCAM-1, ICAM-1, E-selectin and L-selectin was detected on CD34^+^/CD133^+^-stem cells by flow cytometry. The severity of disease was assessed by the Simplified Acute Physiology Score (SAPS) II. Serum concentrations of vascular endothelial growth factor (VEGF) and angiopoietin (Ang)-2 were determined by Enzyme-linked immunosorbent assay. The expression of VCAM-1, ICAM-1, E-selectin and L-selectin by CD34^+^/CD133^+^-stem cells was significantly upregulated in septic patients, and correlated with sepsis severity. Furthermore, high expression of VCAM-1 by CD34^+^/CD133^+^-stem cells revealed a positive association with mortalitiy (p<0.05). Furthermore, significantly higher serum concentrations of VEGF and Ang-2 were found in septic patients, however none showed a strong association with survival. Our data suggest, that VCAM-1 upregulation on CD34^+^/CD133^+^-stem cells could play a crucial role in their homing in the course of sepsis. An increase in sepsis severity resulted in both and increase in CD34^+^/CD133^+^-stem cells and VCAM-1-expression by those cells, which might reflect an increase in need for vascular repair.

## Introduction

In the course of sepsis, altered endothelial function appears in macro- and microcirculation and contributes significantly to the development of multiple organ failure [1,2]. Reconstitution of the endothelial layer can be initiated by the recruitment of vascular progenitor cells [3–6]. It was demonstrated, that CD34+/CD133+-stem cells in septic patients contain a distinct amount of additional KDR (vascular endothelial growth factor receptor 2) expression—as indicative of endothelial progenitor cells (EPC) -[7] and are increasingly mobilized in sepsis compared to non-septic ICU patients and healthy individuals[8]. Furthermore the increased mobilization of CD34^+^/CD133^+^-stem cells in sepsis correlated with survival[8].

The recruitment of vascular progenitor cells to inflammatory endothelial tissue is a complex process and involves a coordinated multi-step-process including mobilization, chemotaxis, homing and paracrine interaction with the resident cells [9]. Homing of endothelial progenitors, for example, to the target tissue has been shown to be influenced by various chemokines, cytokines, adhesion molecules and proteases [10–13]. Certain adhesion molecules, which play a critical role in leucocyte homing, were also identified as key regulators of transendothelial migration of EPC [14]. In that respect, E-selectin and P-selectin have been demonstrated as mediators of leucocyte rolling which are induced on EPC by the stromal cell factor 1 (SDF-1) and promote EPC homing to sites of critical ischemia [15]. Furthermore, Vascular Cell Adhesion Molecule-1 (VCAM-1) is needed for EPC adhesion to fibroblasts from arthritic tissue [16]. Nevertheless, the exact molecular mechanisms of vascular progenitor cell homing especially to sites of vascular inflammation in septic patients are still poorly understood.

Assuming, that homing of vascular progenitor cells in sepsis involves various mediators that recruit them to activated endothelium in response to a damage-induced inflammation, we set out to determine in septic patients which adhesion molecules are expressed by CD34^+^/CD133^+^-stem cells—containing vascular progenitors [17,18]—and could therefore be involved in the CD34^+^/CD133^+^-stem cell-driven repair process. Furthermore, we wanted to analyse, if the upregulation of adhesion molecules by CD34^+^/CD133^+^-stem cells is associated with survival or mortality of septic patients.

## Material and methods

### Subjects

For this study, we enrolled over a 3-year period 30 septic patients from the Intensive Care Unit (ICU) of the University Medical Center Mannheim at admission to the ICU or within 48 hours after onset of sepsis. Selected patients met the diagnostic criteria for sepsis of the American College of Chest Physicians/Society of Critical Care Medicine [19]. The severity of sepsis was determined by the Simplified Acute Physiology Score (SAPS II) [20], and mortality was specified by death occurring within 28 days after diagnosis. According to our previous publication [8], we used the following exclusion criteria: cardiogenic or hemorrhagic shock, chronic obstructive pulmonary disease, isolated acute respiratory distress syndrome, absence of mechanical ventilation, and use of statins, angiotensin-converting enzyme inhibitors, activated protein C, and hydrocortisone. We recruited 10 patients from the ICU who required mechanical ventilation and healthy volunteers from our laboratory staff as control groups. ICU controls did not meet the criteria for sepsis, septic shock, or systemic inflammatory response syndrome. 15 healthy subjects served as controls. The Ethics Committee of the University of Heidelberg has approved this study and written informed consent was obtained from all study subjects.

### Blood sampling

Blood (20 mL) from septic patients was obtained within 24hrs after onset of sepsis and from ICU controls within 24hrs after admission to the ICU.

### Flow cytometry

Peripheral blood mononuclear cells (PBMC) were isolated by Ficoll gradient centrifugation (Amersham Biosciences, Freiburg, Germany). Cell-surface antigens expression was quantified by Fluorescence Activated Cell Sorting (FACS) analysis as described previously [8]. The following anti-human monoclonal antibodies have been used: PE-conjugated CD133 (Miltenyi Biotec, Bergisch-Gladbach, Germany), PerCP-conjugated CD34 (BD Biosciences, Heidelberg, Germany), and either FITC-conjugated VCAM-1/CD106, FITC-conjugated ICAM-1/CD54, FITC-conjugated E-selectin/CD62E and FITC-conjugated L-selectin/CD62L. Flow cytometry was executed on a FACSCalibur flow cytometer (BD Biosciences) and data analysis was performed using WinMDI 2·8 software (Scripps Research Institute, La Jolla, CA). CD34^+^/CD133^+^-stem cell counts are expressed as percentage of total PBMC in each patient or control.

### Enzyme-linked immunosorbent assay

The serum concentrations of Vascular Endothelial Growth Factor (VEGF) and Angiopoietin-2 (Ang2) were measured using enzyme-linked immunosorbent assay kits (R&D Systems, Wiesbaden-Nordenstadt, Germany) in triplicate samples according to the manufacturer’s instructions.

### Statistical analysis

For our statistical analyses we used both parametric and nonparametric methods. All presented data are displayed as mean ± SEM. We examined all data for normal and non-Gaussian distribution by the Kolmogorov-Smirnov test. For comparison among normally distributed groups, one-way ANOVA, followed by pairwise multiple comparison (Student-Newman-Keuls method) was used. For non-normally distributed data, the nonparametric Kruskal-Wallis test followed by an all pairwise multiple comparison (Dunnett’s method) was used. Since the mean age in the group of healthy controls was significantly lower than in the two patient groups, we adjusted for age to avoid confounders. For comparative analysis of survival in the group of septic patients, we used Student’s *t*-test and U test. Logistic regression analysis was performed to predict survival probability from CD34^+^/CD133^+^-stem cell numbers and from adhesion molecule expression by CD34^+^/CD133^+^-stem cells. All assessed variables have been put under correlation analyses (Pearson/Spearman). P<0.05 was considered statistically significant. We used the SAS system (version 8.2) for all the above mentioned aspects. The logistic regression curve has been generated with Minitab 18 (Friedrichsdorf, Germany).

## Results

### Patient population

The relevant clinical data of study participants regarding age, gender, mortality, SAPSII score, type of infection, White Blood Cell (WBC) count and procalcitonin (PCT) are summarized in Table 1.

**Table 1:** Clinical characteristics of patients and controls. Clinical data of study participants for age, gender, mortality, Simplified Acute Physiology Score (SAPS) II score, type of infection, white blood cell (WBC) count and procalcitonin (PCT) refer to the time point of blood sampling. The mean age in the group of healthy controls was significantly lower compared to the patient groups (p = 0,0001). There was no statistical difference in mean age between the two patient groups (p = 0,065).

	Group		
characteristics	healthy controls	ICU controls	septic patients
Number of subjects	15	10	30
Age (years)			
median (range)	32 (26–63)*	46 (21–80)	67 (39–84)
Gender			
male % (total)	47 (7)	60 (6)	53 (16)
female % (total)	53 (8)	40 (4)	47 (14)
Mortality < 28 days (%)		0	46,67
SAPS II score (range)		31,8 (6–69)	47,9 (16–78)
Type of infection			
*Pneumonia*			*15*
*Peritonitis*			*11*
*Meningitis*			*2*
*Pancreatitis*			*1*
*Intracranial Bleeding*			*1*
WBC (x10^9^/L)		10.4	17,2
PCT (ng/ml)		2,3	10,9

*ICU*: intensive care unit; *SAPSII*: Simplified Acute Physiology Score II; *WBC*: white blood cell count; *PCT*: procalcitonin

“*” marks a statistically significant difference (p<0,05).

### CD34^+^/CD133^+^-stem cells correlate with survival

**Fig 1:**
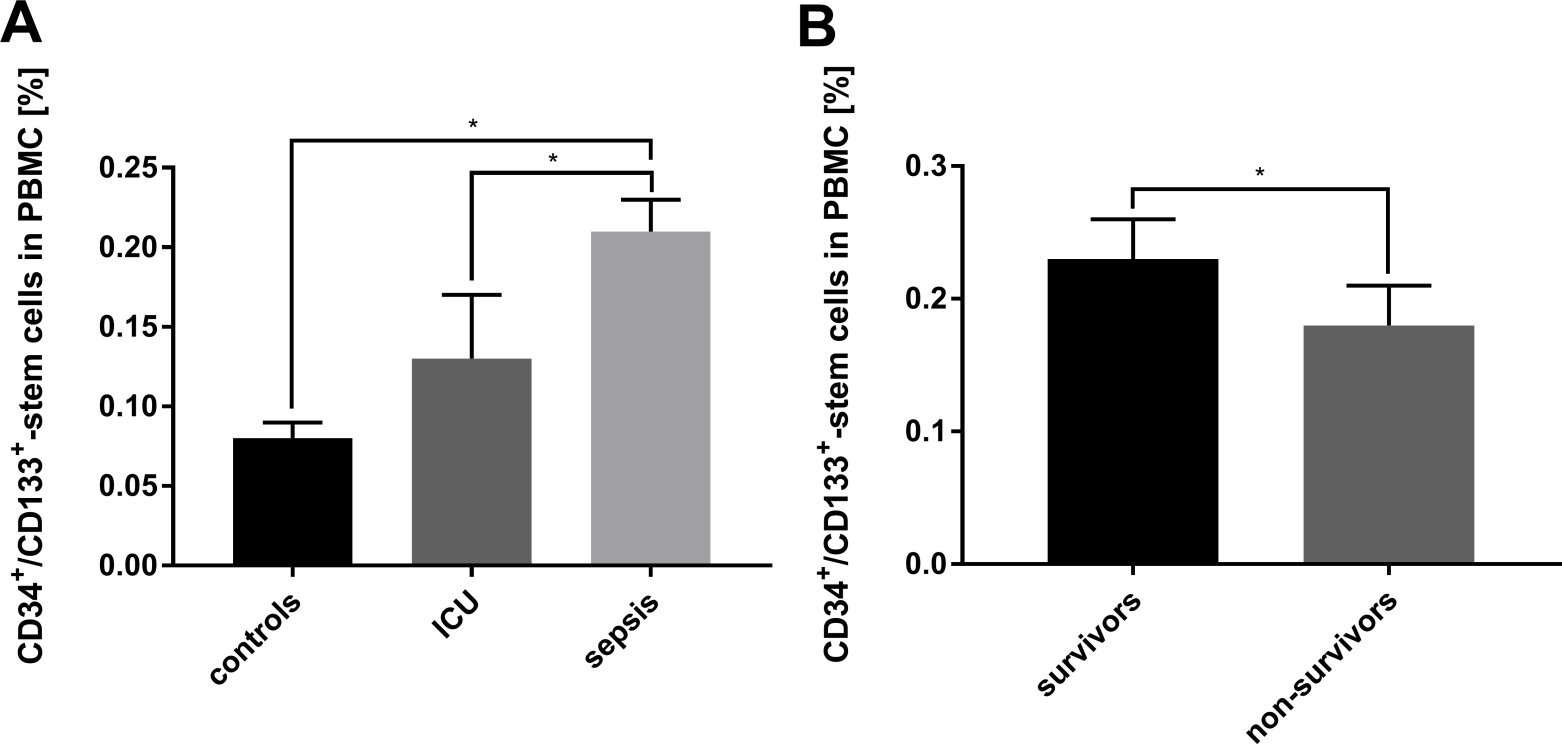
Percentage of circulating CD34^+^/CD133^+^-stem cells. (**A**) FACS analysis of CD34/CD133-positive cells in the peripheral blood mononuclear cell (PBMC) fraction of healthy volunteers (n = 15), non-septic intensive care unit (ICU) patients (n = 10) and septic patients (n = 30). Significant differences were found between the three groups. (**B**) FACS analysis of CD34/CD133-positive cells of septic patients, stratified for survival. Data are given as mean ± SEM; * p*<*0.05 was considered to be statistically significant.

The percentage of CD34^+^/CD133^+^-stem cells was significantly increased by 61% in septic patients compared to ICU and by 162% compared to healthy controls. CD34^+^/CD133^+^-stem cells numbers in ICU patients were also significantly increased over healthy controls (Fig 1). Within the group of septic patients, numbers of CD34^+^/CD133^+^-stem cells in sepsis survivors were increased by 35% compared to non-survivors, however, this difference was not significant (Fig 1). Septic patients with an age below 55 years (n = 7) had very low levels of circulating CD34^+^/CD133^+^-stem cells compared to older patients (data not shown). When excluding these 7 patients in the statistical analysis—since age has a negative effect on vascular progenitor cell numbers—the CD34^+^/CD133^+^-stem cells in sepsis-survivors were significantly increased by 76% compared to non-survivors (Mean ± SD: 0,29 ± 0,13% vs. 0,17 ± 0,13%, p = 0,034).

### Adhesion molecules expressed by CD34^+^/CD133^+^-stem cells

**Fig 2:**
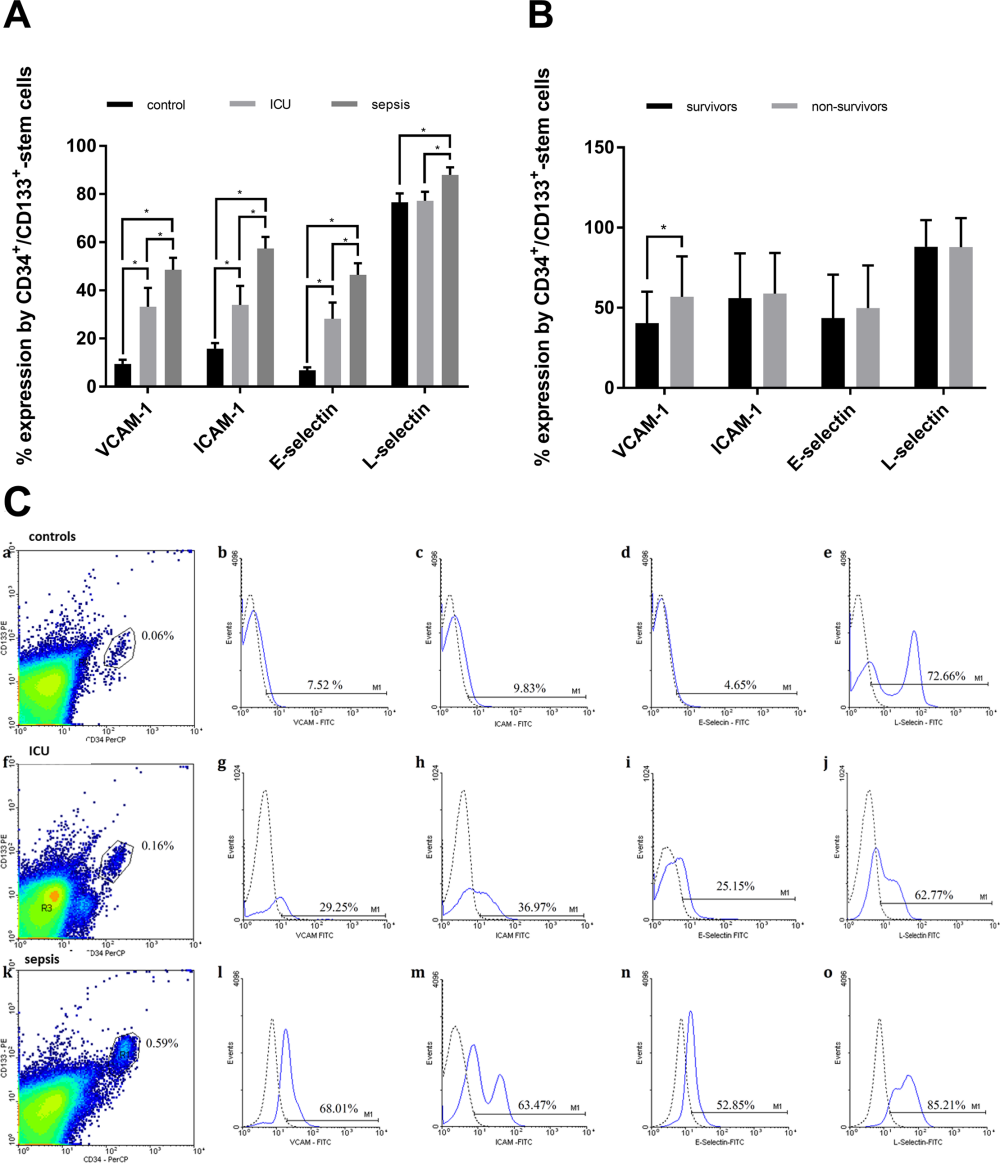
Upregulation of adhesion molecules by CD34^+^/CD133^+^-stem cells. (**A**) FACS analysis of the expression of VCAM-1, ICAM-1, E-selectin and L-selectin by CD34/CD133—positive cells in the peripheral blood mononuclear cell (PBMC) fraction of healthy volunteers (n = 15), non-septic intensive care unit (ICU) patients (n = 10) and septic patients (n = 30). Significant differences were found between the three groups and marked with *. (**B**) shows [%] expression of adhesion molecules by CD34^+^/CD133^+^-stem cells. Logistic regression analysis revealed a significant negative association of VCAM-1 expression by CD34^+^/CD133^+^-stem cells and sepsis survival probability, which is marked with * in the figure. (**C**) FACS analysis data representative for each investigated group: healthy volunteers (**a-e**), ICU controls (**f-j**) and septic patients (**k-o**). Dot plots show PBMC stained with anti-CD34 and anti-CD133 monoclonal antibodies; histograms show the percentage of VCAM-1, ICAM-1, E-selectin and L-selectin expression in the population of CD34/CD133-positive cells. The dotted line in histograms represents the negative control. * significant differences (p<0,05). *FITC*, fluorescein; *PE*, phycoerythrin; *PerCP*, peridinin chlorophyll protein complex. VCAM-1, vascular adhesion molecule-1; ICAM-1, intercellular adhesion molecule-1.

**Fig 3:**
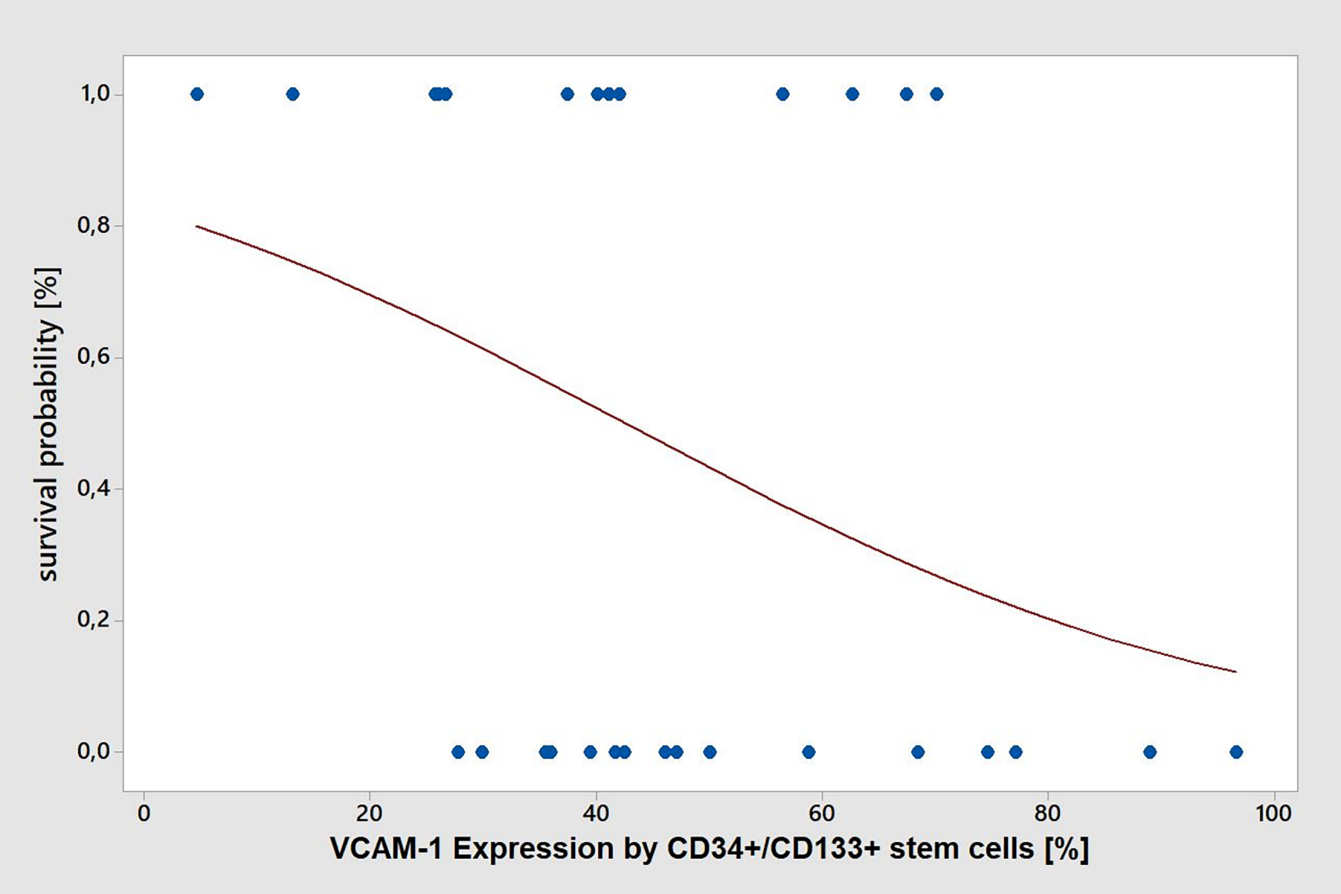
Logistic regression analysis of VCAM-1 expression by CD34^+^/CD133^+^-stem cells and sepsis survival. Binary logistic regression analysis revealed a significant negative association of VCAM-1 expression by CD34^+^/CD133^+^-stem cells with sepsis survival probability (p = 0.05). Equation of regression-line: **p(100) = exp(1,56 - 0,0366 VCAM-1 expression)/(1 + exp(1,56 - 0,0366 VCAM-1 expression)).** *p(100)*, probability of survival, *VCAM-1*, vascular adhesion molecule-1.

The expression of VCAM-1 by CD34^+^/CD133^+^-stem cells from septic patients was significantly increased by 46% compared to CD34^+^/CD133^+^-stem cells from ICU or by 47% compared to CD34^+^/CD133^+^-stem cells from healthy controls (Fig 2). Similar results were found for Intercellular Adhesion Molecule-1 (ICAM-1) and E-selectin. However, differences in the expression of L-selectin between septic patients and ICU patients or healthy controls were less prominent (14% increase of CD34^+^/CD133^+^-stem cell counts in septic patients compared to ICU patients) (Fig 2). Logistic regression analysis revealed an inverse association between expression of VCAM-1 and survival probability in the sepsis group (p = 0.05). For the other adhesion molecules, no such significant difference could be found (Fig 2). In addition, there was no significant association between patient age and VCAM-1 expression by CD34^+^/CD133^+^-stem cells (Fig 3).

### Sepsis severity correlates with mortality and with expression of VCAM-1 and E-selectin by CD34^+^/CD133^+^-stem cells

Severity of sepsis was assessed by SAPS II scoring. The mean SAPS II score was significantly higher in the non-survivor than in the survivor group by 47% (Mean ± SEM: 57,8 ± 3,7 vs. 39,2 ± 4,1; p = 0,0024). Furthermore, a significant correlation between SAPS II and the expression of VCAM-1 (r = 0.61, p = 0.01) and E-selectin (r = 0.59, p = 0.02) by CD34^+^/CD133^+^-stem cells of sepsis survivors could be detected, but not of non-survivors. There were no significant differences in PCT and WBC count between survivors and non-survivors of sepsis.

### Serum concentrations of VEGF and Ang-2 are elevated in septic patients

**Fig 4:**
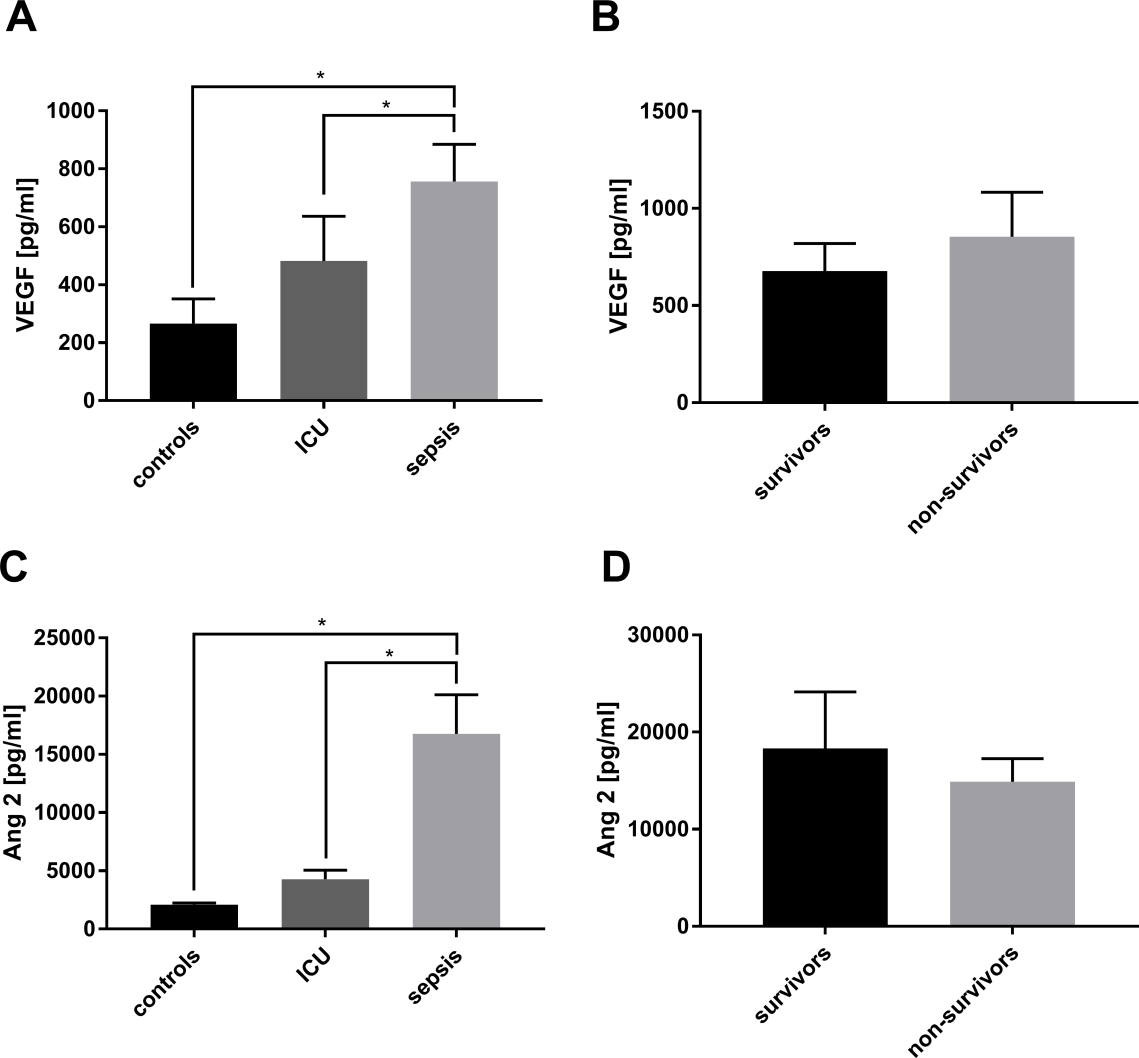
Upregulation of mobilizing growth factors in serum. (**A**) Vascular endothelial growth factor (VEGF) and (**C**) angiopoietin (Ang)-2 concentrations were detected in the serum of healthy volunteers (n = 15), non-septic intensive care unit (ICU) patients (n = 10) and septic patients (n = 30). (**B** and **D**) The group of septic patients was also divided by survival and serum concentrations of both VEGF and Ang2 are indicated. The results are expressed as mean simple linear regression ± SEM; * p*<*0.05 was considered to be statistically significant.

Serum VEGF and Ang2 concentrations were significantly higher in septic patients compared with ICU and healthy controls (Fig 3). Within the group of septic patients, the serum concentration of VEGF and Ang-2 between survivors and non-survivors was not significantly different (Fig 4).

For the entire study population, a significant correlation between CD34^+^/CD133^+^-stem cell numbers and serum levels of VEGF (r = 0.31, p = 0.03) and Ang-2 (r = 0.32, p = 0.02) was observed, indicating that high numbers of CD34^+^/CD133^+^-stem cells are correlated to high serum levels of VEGF and Ang-2. The CD34^+^/CD133^+^-stem cell numbers in septic patients or in the survivor/non-survivor subgroups was not correlated with serum levels of VEGF or Ang-2.

## Discussion

In this study, we demonstrate that CD34^+^/CD133^+^-stem cells, which have been described to be rich in vascular progenitor cells [7], exhibited a high expression of the adhesion molecule VCAM-1 in septic patients, which revealed a positive association with sepsis mortality. Additionally, we confirmed our previous findings of an increased mobilization of CD34^+^/CD133^+^-stem cells in septic patients compared with non-septic ICU patients and healthy individuals and of a respective correlation between stem cell numbers and survival [8].

The recruitment of vascular stem cells to sites of endothelial injury and dysfunction is mediated by several adhesion molecules, which are essential for binding of those cells to the endothelial layer or to the extracellular matrix [10–12,21]. Endothelial progenitor cells, for example, express PSGL-1 (P-selectin-glycprotein-ligand) and are recruited to activated endothelial cells *via* P- and E-selectin [11,22]. In a murine endotoxemia model, VCAM-1 was shown to be involved in endotoxin-induced leukocyte sequestration [23]. As vascular stem cells have been shown to beneficially influence the outcome of sepsis, it seems likely, that homing of those cells during sepsis is intensified [8]. However, the expression of adhesion molecules by vascular progenitors in the course of sepsis has not been investigated in clinical studies so far. Experimental data show, that the inflammatory cytokine TNF-α can induce a similar degree of surface adhesion molecule upregulation in endothelial progenitor cells as it does in mature endothelium [24]. Since high amounts of pro-inflammatory cytokines are present in the circulation in the course of sepsis, the expression of adhesion molecules by vascular stem cells of septic patients is probably increased, which is crucial for the firm adhesion and homing of those cells to the inflamed endothelium. In accordance to that hypothesis, we could show in our study, that CD34^+^/CD133^+^-stem cells from septic patients exhibited significantly higher expressions of the adhesion molecules VCAM-1, ICAM-1, E-selectin and L-selectin compared to ICU and healthy controls. Among these, VCAM-1 was significantly associated with sepsis mortality. Logistic regression analysis indicated, that septic patients with high expression of VCAM-1 by CD34^+^/CD133^+^-stem cells had a higher probability to die compared to patients with lower expression. It has been shown, that an increase in soluble forms of adhesion-molecules and an increased expression of those on the endothelium is present in inflammatory conditions such as sepsis, and are associated with mortality and multi-organ failure [25–27]. Our study now shows, that VCAM-1 expression is also increased specifically on CD34^+^/CD133^+^-stem cells during sepsis, which has not been investigated before. This increased expression was correlated to sepsis severity and associated with patient mortality. The increase in sepsis severity might reflect an increasing need for CD34^+^/CD133^+^-stem cell-based vascular regeneration facilitated by an improved homing via VCAM-1. This hypothesis, however, is associative and should be addressed in future studies. Our current work does not provide mechanistic insights into the proposed importance of VCAM-1 expression on CD34^+^/CD133^+^-stem cells for homing processes in sepsis.

Besides the higher expression of adhesion molecules on CD34^+^/CD133^+^-stem cells, we could additionally detect a significant increase of the numbers of those stem cells in septic patients compared to non-septic ICU controls. This finding is in concert with the increase of VCAM-1 expression on CD34^+^/CD133^+^-stem cells in septic patients and might again be interpreted as a measure to counteract vascular damage as present in sepsis. There is evidence, that CD34^+^- stem cells show distinct regenerative capacities, consistent with vascular repair as attributed to classical endothelial progenitors [28,29]. We could in our previous studies already demonstrate that septic patients exhibit increased levels of circulating endothelial progenitor cells themselves, using FACS analysis[8]. Other groups have confirmed these results in human studies using the flow cytometry to analyze and count endothelial progenitors [30–32]; besides human studies, Luo et al. showed an increase of circulating vascular stem cells in the early phase of endotoxemia induced sepsis in pigs with a peak after 72 hours [33]. However, studies based on the isolation of vascular progenitor cells by colony forming assays instead of flow cytometry did not show a significant increase in progenitor cell mobilization in sepsis [34,35]. The differences in methodology might explain the differences in demonstrated vascular stem cell number changes in sepsis. In our current study the detected an increase in CD34^+^/CD133^+^-stem cell numbers in septic patients using flow cytometry indicates, that there seems to be a distinct positive impact of sepsis on CD34^+^/CD133^+^-stem cell mobilization, which might reflect the need for vascular repair.

Mobilizing vascular stem cells from the bone marrow during inflammatory diseases is a multi-faceted process, not only regulated by surface bound adhesion molecules, but also by a variety of paracrine factors and downstream signaling cascades [36]. It has been demonstrated, that the mobilization and general function of vascular stem cells like migration and proliferation are improved by circulating growth factors such as VEGF and Ang2 [37–40]. With regards to sepsis, a correlation between increased plasma VEGF levels and severity of Multi-Organ-Dysfunction-Syndrome (MODS) has been reported by several groups [41,42]. Based on those studies, VEGF was discussed to influence sepsis-associated capillary leakage [41] and be an important determinant of sepsis morbidity and mortality[43]. However, a recent study by Besnier et al. could show, that anti-VEGF therapy in septic mice did not influence survival [44]. Ang2 serum levels are also increased in septic patients, which is associated with a poor prognosis, as Ang2 is destabilizer of mature endothelial linings and a promoter of vascular leakage [45]. In this study, we confirmed the increase of VEGF serum levels in septic patients, but did not observe significant serum level differences between survivors and non-survivors similar to our previous work [8]. Comparable results were found for Ang-2. The clinical impact of the combined increase in VEGF and Ang2- serum levels on vascular stem cell functions like homing and trans-endothelial migration in septic patients remains to be determined in future studies. However, we could demonstrate a correlation between CD34^+^/CD133^+^-stem cell numbers and both VEGF and Ang2 serum levels which suggests a distinct role of these growth factors in CD34^+^/CD133^+^-stem cell mobilization in sepsis. Currently, VEGF seems to be rather a marker of sepsis severity instead of being an inductor of sepsis mortality. Therefore, the increase of VEGF in sepsis might even have a beneficial impact on sepsis disease course by promoting endothelial regeneration via CD34^+^/CD133^+^-stem cell mobilization or stabilization of CD34^+^/CD133^+^-stem cell function. Cleary, such a hypothesis will have to be assessed in additional future studies.

Several human and animal studies have demonstrated higher numbers of vascular stem cells in survivors of sepsis [30,31,33]. Contrary to that, the research group of Kung et al. recently saw a negative association between sepsis survival and endothelial progenitor cell numbers [32]. In the light of this controversy, our current data revealed slightly higher CD34^+^/CD133^+^-stem cell counts in sepsis-surviving patients, however, this difference was not significant. Yet, CD34^+^/CD133^+^-stem cell levels in sepsis-survivors older than 54 years were significantly increased over sepsis-non-survivors. We saw in our study, that CD34^+^/CD133^+^-stem cell levels in general were lower in patients with an age below 55 years compared to older patients, which we suspected to be a probable confounding factor in the survival analysis. Therefore, the impact of patient age on sepsis survival associated with vascular stem cells should be selectively assessed in future studies.

In general, the limitation of studies investigating vascular stem cells is the lack of consensus on an unique phenotypic identification of the “endothelial progenitor cell” due to the absence of a unique marker. As mentioned before, cell culturing methods based on colony forming assays or flow cytometry both have their advantages and disadvantages. According to the findings of Herrmann et al. we could demonstrate in our previous work, that bone marrow derived progenitors, with a high expression of CD34 and CD133 are rich in vascular progenitor cells, especially in sepsis [8]. Besides the hematopoetic stem cell marker CD34, we used the progenitor cell marker CD133 to exclude mature endothelial cells from FACS counting. Yet, since the classical definition of EPC requires also an endothelial marker protein like VEGF-R2 (KDR) or CD31, the stem cell populations used in our study include hematopoietic stem cells in their numbers. Another limitation of this study is its cross-sectional design and the subsequent lack of information on CD34^+^/CD133^+^-stem cell numbers during the course of sepsis.

In conclusion, we have demonstrated here for the first time that CD34^+^/CD133^+^-stem cells in the clinical setting of sepsis exhibit a high expression of VCAM-1 and that this expression is associated with patient survival. Furthermore, our results confirm our previous observations of an increased mobilization of vascular stem cells in septic patients and their correlation with survival. Based on that, we suggest, that VCAM-1 signaling appears to play a distinct role in CD34^+^/CD133^+^-stem cell homing in the course of sepsis. Further studies will be needed to intensify our understanding of the biologic pathways involved and to deduce possible therapeutic implications to improve sepsis outcome.

## Abbreviations

Ang2: Angiopoietin-2
EPC: Endothelial Progenitor Cells
FACS: Fluorescence Activated Cell Sorting
ICAM-1: Intercellular Adhesion Molecule-1
ICU: Intensive Care Unit
MODS: Multi Organ Dysfunction Syndrome
PBMC: Peripheral Blood Mononuclear Cells
PCT: Procalcitonin
SAPSII: Simplified Acute Physiology Score II
SDF: Stromal Cell Derived Factor
VCAM-1: Vascular Cell Adhesion Molecule-1
VEGF: Vascular Endothelial Growth Factor
WBC: White Blood Cell Count
